# Are immigrant populations aware about their oral health status? A study among immigrants from Ethiopia

**DOI:** 10.1186/1471-2458-9-205

**Published:** 2009-06-26

**Authors:** Avi Zini, Yuval Vered, Harold D Sgan-Cohen

**Affiliations:** 1 Department of Community Dentistry, Hebrew University-Hadassah School of Dental Medicine, Jerusalem, Israel

## Abstract

**Background:**

Evidence from Western countries indicates that there are fundamental discrepancies between self-perceived illness of immigrants and the provision of health care, according to the Western bio-medical health service model. These need to be understood in the planning and implementation stages of public health care programs for new immigrants. The objectives of the present study were to investigate self-perceived versus clinically diagnosed dental and periodontal health status among immigrants from Ethiopia.

**Methods:**

During 2004–2005, dental and periodontal health status was recorded among 340 Ethiopian immigrants, utilizing the DMFT and CPI indices. Additionally, participants were interviewed using a questionnaire which included perceived dental and periodontal health status. Sensitivity and specificity levels of this perception were calculated and compared with the published scientific literature.

**Results:**

Regarding dental caries, according to the three operational cut-off points, sensitivity ranged from 70% to 81%, and specificity ranged from 56% to 67%. Regarding periodontal status, 75% of the subjects clinically diagnosed with periodontal pockets self-perceived a "bad" health status of gums (sensitivity) and 54% of the subjects diagnosed without periodontal pockets, reported a "good" health status of gums (specificity). These indications of perception levels were higher than a previous study conducted among native born Israelis.

**Conclusion:**

Minority ethnic groups should not be prejudicially regarded as less knowledgeable. This is illustrated by the unexpected high level of oral health status perception in the present population. Oral health promotion initiatives among immigrants should be based upon optimal descriptive data in order to accomplish the inherent social commitment to these diverse populations.

## Background

Caries and periodontal diseases, the two most common oral pathologies, affect all populations throughout life [1]. Indigenous poor, immigrants, racial and ethnic minorities, and medically compromised populations, are often those who suffer the worst oral health [1,2].

Evidence indicates that immigrants and minority ethnic groups should be regarded as "whole populations at risk" on the verge of oral health deterioration. People crossing national and cultural frontiers often originate from populations with disease patterns, health behaviors and health care measures different from those at their destination. Upon entering a "Western" society, immigrants commonly experience a social "culture shock", which involves social, cultural, environmental, and psychological determinants. The stress of migration can lead to depression, sadness, lack of self-confidence, personal and family crises, low utilization of health services and unfavorable health behavior [3-7].

The State of Israel absorbs immigrants from many countries of the world, including Ethiopia. Developing African countries are often characterized by widespread poverty, scarce organized health promotion programs, lack of optimal water fluoridation, and low extrinsic sugar consumption [8,9]. Israel has a rich tradition and literature about immigrants, their health, their health perception and their needs. Shuval has described migration as a potentially stressful experience, derived from leaving one cultural milieu and entering another. She has suggested that immigration to Israel has had major health effects on Israeli society, including an influence on stress and patterns of disease in the population [10]. Israeli research has described tuberculosis, menopause, aging, cancer and a wide spectrum of other medical conditions among immigrants. Differences have been shown in health patterns and behavior, perception of and coping with disease, utilization of health services and barriers to health care and other health-related social and cultural issues [11-14]. In a study of tuberculosis among Ethiopian immigrants to Israel, Chemtob et al [11] have noted that anthropological and sociological differences among immigrant populations are often neglected. They have noted that in order to ensure successful and humane absorption of immigrants, host authorities are morally obligated to pay attention to these issues.

Studies conducted in Israel over the last 15 years have indicated a national decrease in caries experience, in accordance to that reported in the industrialized world. This has been attributed to fluoridation of drinking water and almost universal utilization of fluoridated toothpaste [15,16]. However, an oral health cohort study, recently conducted among immigrants to Israel from rural Ethiopia between the years 1999–2005, revealed a deteriorating trend in oral health over this period [17].

Data from many Western countries indicates that there are fundamental discrepancies regarding health beliefs and expectations, definition, self-perceived and expression of illness, and communication, between immigrants and minority ethnic groups and the Western bio-medical health model and services [18,19]. Studies conducted among immigrants to Israel, including Ethiopian immigrant populations, have revealed similar findings [20,21]. Bridging this intercultural gap might have a significant importance for health care promotion among underprivileged groups.

The underlying rationale of the present study is that an optimal understanding of subjective health perception is an imperative component in assessing and planning all community dental health programs, but especially among immigrant and underprivileged populations [22-24]. The objectives were to investigate self-perceived versus clinically diagnosed dental and periodontal health status among immigrants from Ethiopia to Israel.

## Methods

During the summer of 1999 a community of about 4000 immigrants arrived from Quara, a rural region of Ethiopia. Over 1000 were placed in one absorption center near Jerusalem. There was no evidence to indicate any social differences between the distribution of this population and those in other centers. The present study population was therefore considered as representative of the low socioeconomic, relatively homogenous immigrant populations in all of the absorption centers.

The Israeli government supports an adaptation and acclimation period of between six to 18 months, during which time efforts are invested in social, cultural and financial integration. Following this, immigrants are located in different towns across the country, with the aim of continuing their lives as regular citizens. Social welfare services are provided on an on-going basis, where and when needed.

The Hadassah Medical Organization, Human Experimental Ethics Committee (IRB) pre-approved this study (reference # 397 – 28303), in full compliance with the Helsinki Declaration . According to these guidelines, full informed consent (in Amharic) was obtained and documented before commencement of examinees' participation.

During 1999–2000, 792 immigrants in the Jerusalem absorption center aged ≥ 5 years participated in a base-line dental health study, and were examined by two calibrated examiners. In 2004–5, with the assistance of an Ethiopian born study coordinator, 672 subjects out of the previous 792, now dispersed throughout the country, were located [17]. Of this population, 340 were adults, age 18 years or more. These 340 subjects comprised the present study population sample.

Data were collected by one of the previous examiners (Y.V). Dental caries status was recorded employing the DMFT (number of permanent teeth with caries experience – D = decay, M = missing, F = filling) and caries-free indices. Periodontal health status was recorded employing the Community Periodontal Index (CPI). The "percentage of worst" CPI scores (0 = healthy, 1 = bleeding, 2 = calculus, 3 = shallow pockets, 4 = deep pockets) were calculated. The DMFT and CPI indices are recommended by the World Health Organization (WHO) [25].

Explanations were provided via an Ethiopian born interviewer. Subjects were informed of any pathology found and if needed, immediately referred for treatment. Using a portable dental chair, the examinations were conducted with a dental mirror, and an appropriate CPI probe.

The use of questionnaires and interviews is a common component in the collection of diagnostic data and performance of oral health surveys [26-28]. Comparisons of clinical oral health diagnoses versus self-perceived assessments may demonstrate the efficacy of the individual to evaluate personal health status and highlight fields in which self-perceived assessment is precise or imprecise. It has been suggested that the use of questionnaires should be further investigated with reference to self-assessed oral health status of communities [26-28].

In the present study dental awareness was operationally defined as self-perception of oral health status according to the following two questions:

1. "What is your opinion about the health status of your teeth?"

2. "What is your opinion about the health status of your gums?"

Possible answers were "very good", "good", "not so good", and "bad". For research purposes the answers "very good" and "good" were operationally combined and considered to indicate a "good" self-perceived assessment and the answers "not so good" and "bad" indicated a "bad" self-perceived assessment for both teeth and gums.

Study participants were also asked whether they suffer from toothache. Possible answers were "often", "seldom", and "never". For research purposes the answers "often" and "seldom" were operationally combined and considered to indicate a positive answer ("yes") and the answer "never" indicated a negative answer ("no") regarding toothache.

For dental caries status, three cut-off points were chosen for operational definitions:

1. DMFT cut-off-point = 1: Caries-free subjects were operationally defined as "Good" dental status (DMFT score = 0) and subjects with caries were defined as "Bad" dental status (DMFT ≥ 1).

2. DMFT cut-off-point = 4 (according to the mean DMFT in the present study, which was found to be 4.04 ± 5.14): DMFT scores 0–4 = "Good" dental status and DMFT>4 = "Bad" dental status.

3. D (untreated component of DMFT) cut-off-point = 3 (according to the mean D in the present study, which was found to be 2.60 ± 3.25): D score of 0–3 = "Good dental status and D>3 = "Bad" dental status.

Regarding periodontal status, reversible vs. non reversible clinical indicators were chosen for operational definitions: CPI scores = 0 (healthy), 1 (bleeding), and 2 (calculus) – subjects with reversible clinical indicators = "Good" periodontal status, and CPI scores = 3 (shallow pockets), and 4 (deep pockets) – subjects with non reversible clinical indicators = "Bad" periodontal status.

For the association between clinical findings ("gold standard") and self perceived oral health status, sensitivity (the proportion of the individuals who perceived having the disease among those who are clinically diagnosed as having the disease), and specificity (the proportion of the individuals who perceived not having the disease among those who are clinically diagnosed as not having the disease) levels were compared with the published scientific literature.

The statistical processing was performed by SPSS 15.0 software. A statistical test was considered significant when p < 0.05.

## Results

The study population comprised of 145 (43%) males and 195 (57%) females. Thirty one subjects comprised the 18 yr-old group, 95 subjects comprised the 35–44 yr-old group and 65 subjects comprised the 51+ yr-old group.

According to the clinical examination, 219 subjects (64%) were found to have experienced dental caries and 36% were caries-free. Mean whole population DMFT was found to be 4.04 ± 5.14 (D = 2.60 ± 3.25, M = 1.27 ± 2.56, F = 0.17 ± 0.94). These data indicate that only 4.2% of the existing caries had been treated (the F component of the DMFT index). For ages 18, 35–44 and 51+ years these levels had deteriorated and were: 6.7%, 1.6% and 0.6%, respectively [17]. The DMFT does not differentiate lesions by their severity and it is possible that the non-treated teeth had deep neglected carious lesions.

One hundred and twenty four subjects (36%) demonstrated periodontal pockets (shallow or deep pockets), and 216 subjects (64%) demonstrated no periodontal pockets (healthy, bleeding or calculus at worst). Among 35–44 year-olds 15.3% demonstrated deep pockets. This status deteriorated with age and reached 26.8% with deep pockets among subjects over the age of 51 years [17].

For self perceived status of teeth, 192 subjects (57%) reported a "bad" health status. For self perceived status of gums, 191 (56%) reported a "bad" health status. For tooth ache, 203 subjects (60%) reported that they suffered from tooth ache.

Stratification by gender and age revealed no statistically significant differences.

Data regarding the sensitivity and specificity values for different cut-off points are summarized in Table 1. For DMFT = 1 cut-off point, sensitivity was 0.70 and specificity was 0.67. For DMFT = 4 cut-off point, sensitivity was 0.81 and specificity was 0.56. For D = 3 cut-off point, sensitivity was 0.77 and specificity was 0.57.

**Table 1:** Self-perceived and clinically diagnosed dental health status of 340 study subjects (2004–2005).

Clinical Examination	Self Perceived Dental Health Status	
	"Good"	"Bad"
"Good"		
DMFT = 0(caries-free)*	81 **(67%)**	40 (33%)
DMFT = 0–4*	125 **(56%)**	100 (44%)
D = 0–3*	115 **(57%)**	88 (43%)
		

"Bad"		
DMFT≥1*	67 (30%)	152 **(70%)**
DMFT>4*	22 (19%)	93 **(81%)**
D>3*	32 (23%)	105 **(77%)**

* p < 0.001, Fisher's Exact Chi-square test

Cut-off points:

DMFT = 1: Sensitivity = 70%, Specificity = 67%

DMFT = 4: Sensitivity = 81%, Specificity = 56%

D = 3: Sensitivity = 77%, Specificity = 57%

Regarding periodontal status, periodontal health was operationally defined as no periodontal pockets (CPI scores 0, 1, and 2 – reversible indicators), vs. periodontal pockets (CPI scores 3 and 4 – non reversible indicators). As presented in Table 2, 92 out of 124 subjects (75%) clinically diagnosed with periodontal pockets, reported a "bad" health status of gums (sensitivity) and 117 out of 216 subjects (54%) clinically diagnosed without periodontal pockets, reported a "good" health status of gums (specificity).

**Table 2:** Self-perceived and clinically diagnosed periodontal health status of 340 study subjects (2004–2005).

Clinical Examination	Self Perceived Periodontal Health Status	
	"Good"	"Bad"
"Good"		
Without Periodontal Pockets*	117 **(54%)**	99 (46%)

"Bad"		
Periodontal Pockets*	32 (25%)	92 **(75%)**

* p < 0.001, Fisher's Exact Chi-square test

Sensitivity = 75%, Specificity = 54%

## Discussion

Similar to other Westernized countries, Israel has experienced an ongoing influx of immigrants. International data have revealed that immigrants and minority ethnic groups should be regarded as prone towards oral health deterioration [5,29-31]. In the report on Oral Health in America, the U.S. Surgeon General has called for additional efforts to identify and reduce oral health disparities [2]. There is a need to understand how immigrant groups assess their oral health. Examining adults' perception of their oral health status provides important information that could contribute towards public oral health promotion [2,5,18,19,23,24,26]. The present study, first of its kind in Israel, offered a unique opportunity to examine oral health awareness among immigrants from Ethiopia, according to self-perception as compared to clinical diagnosis.

Data from the third National Health and Nutrition Examination Survey (NHANES III, 1988–1994) performed in the United States, can be used to obtain estimates of perceived oral health status among the civilian, noninstitutionalized U.S. population [23]. Approximately one-third of Americans 20 years of age or older described the condition of their natural teeth as either "poor" or "fair". The remaining two-thirds reported the condition as being "good", "very good" or "excellent". Among these adults, nearly two percent reported requiring relief from pain.

In a U.S. study conducted among a refugee population, 49% of the participants rated their dental health as "fair" and "poor" [18]. Additional studies among different minority groups in the USA and the U.K. demonstrated that 35% to 50% of the participants rated their oral health as "fair" and "poor" [19,32-34].

In the present study, 57% of the participants reported a "bad" health status of their teeth, 56% reported a "bad" health status of their gums, and 60% reported that they suffered from tooth ache. This high level of reported tooth ache may have been due to the extremely low level of treated caries and possibly deep neglected lesions.

Regarding the association between clinical examination findings and perceived oral health status, among the NHANES III individuals with 3 or more teeth with untreated decay, 39% described the condition of their natural teeth as "poor" [23]. Nearly 75% of individuals with no untreated decay reported the condition of their natural teeth to be "good" (38%), "very good" (23%), or "excellent" (14%).

It has been noted that people in general and immigrant and minority groups in particular, are often unable to recognize whether they are affected by dental and periodontal diseases [18,19,26,29-34]. In our study, according to the three operational cut-off points, sensitivity ranged from 70% to 81%, and specificity ranged from 56% to 67%. The results of the present study reflect a relatively high level of awareness among this group of Ethiopian immigrants.

The percentage of subjects who reported a "bad" oral health status (56% to 67%) and suffered from tooth ache (60%) is considerably higher than reported in other studies [18,19,23,32-35]. As for the association between clinical exam findings and perceived oral health status, the comparison performed in the present study suggests that perception of health and disease of teeth and gums were high.

The present results, among non-Western immigrants, are contrary to other studies among Western and immigrant populations, which demonstrated lower levels of perception of health and disease [26,18,19,23,32-35]. In a study conducted in Israel [35] among young adults, perceived oral health status was found to be of high specificity (0.83 for dental caries status and 0.83 for periodontal status), but of low sensitivity (0.34 for dental caries status and 0.28 for periodontal status). In the NHANES III study [23], perceived oral health status for dental caries demonstrated high specificity (0.75), but low sensitivity (0.39). The present immigrants had been examined and diagnosed five years earlier in a previous study [17]. It is therefore possible that the subjects are to some extent aware of their dental health situation from the first examination. This previous experience may have influenced the level of self-perceived periodontal and dental health at the present stage.

## Conclusion

National oral health data often focus on clinical findings and inadequately cover social, cultural and environmental factors. These are particularly significant concerning ethnic minority populations [18,19,29,30,32-34]. One of the main achievements of social research is that it gives the patient a voice [22]. Minority ethnic groups should not be prejudicially regarded as less knowledgeable. This is illustrated by the unexpected high level of oral health status perception in the present population. Oral health promotion initiatives among immigrants should be based upon optimal descriptive data in order to accomplish the inherent social commitment to these diverse populations.

## Competing interests

The authors declare that they have no competing interests.

## Authors' contributions

AZ contributed to the statistical analysis and participated in the manuscript presentation. YV conducted the examinations and participated in the design of the study and in the manuscript presentation. HSC participated in the design and manuscript presentation.

## Pre-publication history

The pre-publication history for this paper can be accessed here:


